# Primary care implications of the British Thoracic Society Guidelines for bronchiectasis in adults 2019

**DOI:** 10.1038/s41533-019-0136-8

**Published:** 2019-06-27

**Authors:** Kevin Gruffydd-Jones, Duncan Keeley, Vikki Knowles, Ximena Recabarren, Alex Woodward, Anita L. Sullivan, Michael R. Loebinger, Karen Payne, Alex Harvey, Lizzie Grillo, Sally A. Welham, Adam T. Hill

**Affiliations:** 1 Box Surgery, Wiltshire, UK; 2 Thame Health Centre, Thame, UK; 3 Virgin Care, Farnham Hospital, Farnham, UK; 4 Respiratory Department, Royal Infirmary of Edinburgh, Edinburgh, UK; 5 Leicestershire Partnership NHS Trust, Leicester, UK; 6 Department of Respiratory Medicine, University Hospitals Birmingham NHS Foundation Trust (Queen Elizabeth Hospital), Birmingham, UK; 7 Host Defence Unit, Department of Respiratory Medicine, Royal Brompton Hospital and Harefield NHS Foundation Trust, London, UK; 8 Glenfield Hospital, Leicester, UK; 9 Department of Clinical Sciences, Brunel University London, London, UK; 10 Royal Brompton Hospital, London, UK; 11 British Thoracic Society, London, UK; 12 Respiratory Medicine, Royal Infirmary of Edinburgh, and University of Edinburgh, Edinburgh, UK

**Keywords:** Respiratory tract diseases, Respiratory signs and symptoms

## Abstract

The British Thoracic Society (BTS) Guidelines for Bronchiectasis in adults were published in January 2019, and comprise recommendations for treatment from primary to tertiary care. Here, we outline the practical implications of these guidelines for primary care practitioners. A diagnosis of bronchiectasis should be considered when a patient presents with a recurrent or persistent (>8 weeks) productive cough. A definitive diagnosis is made by using thin-section chest computed tomography (CT). Once diagnosed, patients should be initially assessed by a specialist respiratory team and a shared management plan formulated with the patient, the specialist and primary care teams. The cornerstone of primary care management is physiotherapy to improve airway sputum clearance and maximise exercise capacity, with prompt treatment of acute exacerbations with antibiotics.

## Introduction

Bronchiectasis is a condition where patients have symptoms of persistent or recurrent bronchial sepsis related to irreversibly damaged and dilated bronchi.^1^ The incidence and prevalence of bronchiectasis is rising. Using the Clinical Practice Research Datalink (CPRD) in the United Kingdom, patients 18 and over with a Read code of Bronchiectasis were identified, and the point prevalence in February each year from 2004 to 2013 was measured. This showed that the point prevalence of coded bronchiectasis rose from 351 to 566 per 100,000 in women and from 301 to 486 per 100,000 in males between 2004 and 2013.^2^ A retrospective study using healthcare data from the United States showed a rise in prevalence of 8% between 2001 and 2012, but with a point prevalence of 139/100,000.^3^ The British Thoracic Society (BTS) Guideline for non-cystic fibrosis (non-CF) bronchiectasis was published in 2010^4^ and included children. The BTS commissioned an update to the guidelines in 2013 to take into account any new evidence. The new BTS Guideline covers bronchiectasis in adults and provides recommendations and good practice points based on an updated evidence review.^1^

Systematic electronic database searches of relevant literature were carried out in June 2014 and June 2016, identifying relevant papers published since the previous guideline. In total, 1799 such papers were identified and reviewed by pairs of Guideline Committee members. Appraisal of the evidence was carried out based on Scottish Intercollegiate Guidelines Network (SIGN) methodology, as set out in the BTS Guideline Production Manual.^5^ The reliability of the evidence in each individual study was graded, using the SIGN critical appraisal checklists and levels of evidence assigned to evidence statements, according to the levels of evidence in Table 1.^5,6^ Recommendations made by the Guideline Committee were formulated based on the evidence statements and graded according to the SIGN grading system (Table 2).^6^

**Table 1:** SIGN levels of evidence^5,6^

Grade	Evidence
1++	High-quality meta-analyses, systematic reviews of RCTs or RCTs with a very low risk of bias
1+	Well-conducted meta-analyses, systematic reviews of RCTs or RCTS with a low risk of bias
1−	Meta-analyses, systematic reviews of RCTs or RCTs with a high risk of bias
2++	High-quality systematic reviews of case–control or cohort studies or high-quality case–control or cohort studies with a very low risk of confounding, bias or chance and a high probability that the relationship is casual
2+	Well-conducted case–control or cohort studies with a low risk of confounding, bias or chance and a moderate probability that the relationship is casual
2−	Case–control or cohort studies with a high risk of confounding, bias or chance and a significant risk that the relationship is not casual
3	Non-analytic studies, for example, case reports and case series
4	Expert opinion

*RCT* randomised control trial

**Table 2:** SIGN grades of recommendation^5,6^

Grade	Type of evidence
A	At least one meta-analysis, systematic review or RCT rated as 1++ and directly applicable to the target population *or* a systematic review of RCTs or a body of evidence consisting principally of studies rated as 1+ directly applicable to the target population and demonstrating overall consistency of the results
B	A body of evidence, including studies rated as 2 ++ directly applicable to the target population and demonstrating overall consistency of the results *or* extrapolated evidence from studies rated as 1 ++ or 1+
C	A body of evidence, including studies rated as 2 + directly applicable to the target population and demonstrating overall consistency of the results *or* extrapolated evidence from studies rated as 2 ++
D	Evidence of level 3 or 4 or extrapolated evidence from studies rated as 2+
√	Important practical points for which there is no research evidence, nor is there likely to be any research evidence. The guideline committee wishes to emphasise these good practice points

The composition of the Guideline Committee and the subsequent guideline recommendations were biased towards secondary care. One member of the Guideline Committee (KGJ—lead author of this paper) was from primary care. This paper acknowledges this and with five primary care authors aims to look at the relevance of the guidelines for primary care.

## Initial presentation of bronchiectasis

A diagnosis of bronchiectasis should be suspected when a patient presents with a recurrent or persistent (>8 weeks) cough, with production of purulent or mucopurulent sputum, particularly with the relevant associated risk factors, such as COPD. This diagnosis is particularly likely if the patient has a history of two or more COPD exacerbations per year and a previous positive sputum sample for *Pseudomonas aeruginosa* whilst stable (i.e. not during an exacerbation).^1^

A meta-analysis of six observational studies of 881 patients, mainly based on secondary care, found that the prevalence of bronchiectasis was 54.3% in patients with COPD.^7^ One study conducted in 110 patients from 29 UK general practices showed that the prevalence of bronchiectasis was 29% in patients with COPD.^8^ Patients with bronchiectasis and COPD had a lower FEV_1_-weighted mean difference (WMD)—8%, more frequent exacerbations (WMD 1.54 times more in the previous year) and 7.33 times more likely to have persistent sputum pathogens, especially *P. aeruginosa* than COPD patients without bronchiectasis.^7^

In addition to coincident presentation with COPD, a diagnosis of bronchiectasis should be suspected in patients presenting with persistent cough, who also report difficult-to-treat asthma,^9^ rheumatoid arthritis,^10^ inflammatory bowel disease (IBD),^11^ chronic rhinosinusitis^12^ or the presence of persistent sputum pathogens, especially *P. aeruginosa*.^1^

## History, examination and investigations

If bronchiectasis is suspected in a patient in a primary care setting, a full medical history and examination should be first directed to exclude other causes of cough, such as COPD, lung cancer, asthma and gastro-oesophageal reflux. Persistent basal, coarse lung crackles may be present.

Investigations in the primary care setting should encompass:

- Diagnostic spirometry to look for other/coexistent diagnoses (such as COPD) and may be normal, restrictive or obstructive.
- Sputum microbiology is helpful in identifying the presence of persistent pathogens, especially *P. aeruginosa* (indicating a worse prognosis).
- A chest X-ray should be carried out to help exclude alternative diagnoses, but has limited sensitivity for diagnosing bronchiectasis, especially in mild disease.
- Definitive diagnosis of bronchiectasis is made, using thin-section CT scanning of the chest. It is highly accurate and diagnostic for bronchiectasis, with false-positive and false- negative rates of 1% and 2%, respectively.^13^ Referral for CT will usually be made via a secondary care specialist, although some primary care practitioners may have direct access.

**Fig. 1:**
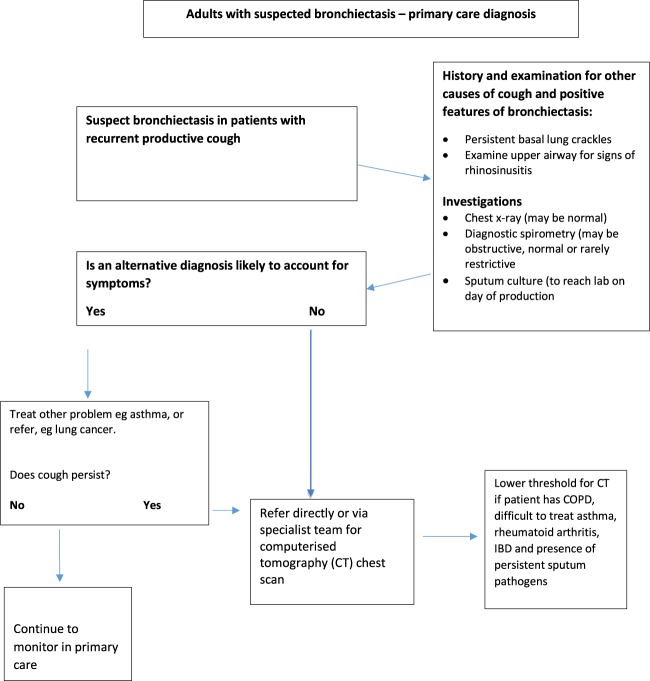
Proposed algorithm for the diagnostic pathway in primary care of patients suspected of having bronchiectasis

Figure 1 shows a suggested diagnostic algorithm by the authors of this article for patients suspected of having bronchiectasis in primary care.

## Assessment and routine

It is recommended that the initial assessment after diagnosis of bronchiectasis is carried out by a specialist respiratory team, where investigations can take place to elucidate any underlying cause and to formulate a treatment plan, which can be shared with the patient, primary care team and community team where appropriate. Single-centre studies suggest that investigations into the underlying cause of bronchiectasis can change management in 5–37% patients.^14^

The underlying causes can include conditions such as allergic bronchopulmonary aspergillosis, immune deficiency, cystic fibrosis (in relevant patients) and non-tubeculous mycobacterial (NTM) disease.^14,15^ Co-morbid conditions should be assessed, including asthma, COPD, gastro-oesophageal reflux disease (GORD), rheumatoid arthritis and inflammatory bowel disease, and treated accordingly.

**Fig. 2:**
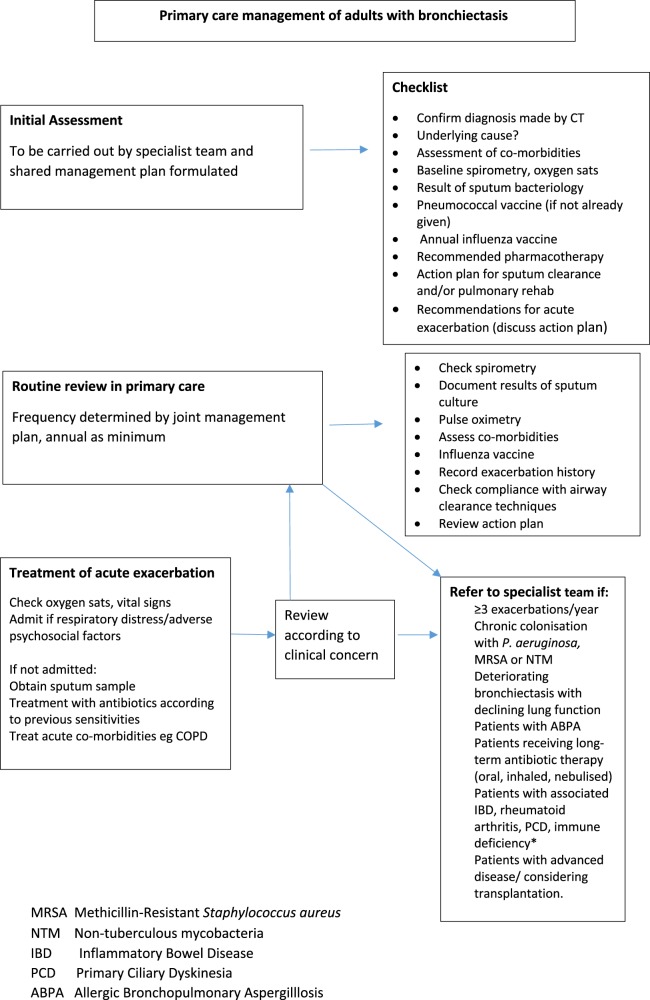
Proposed algorithm for the review and management of patients with bronchiectasis in primary care

The treatment plan should give details of treatment in the stable condition, advice on treatment in the event of an acute exacerbation and arrangements for follow-up (primary care, hospital care and shared care). See Fig. 2 for a suggested checklist as part of the algorithm for review and management of patients wth bronchiectasis in primary care.

## Routine review

Patients at higher risk of future exacerbations should be reviewed in specialist care, where they present with or develop one or more of the following clinical situations:^1^

- They experience three or more exacerbations in 1 year.
- They have chronic *Pseudomonas aeruginosa*, non-tuberculous mycobacteria (NTM) or methicillin-resistant *Staphylococcus aureus* (MRSA) colonisation.
- There is deteriorating bronchiectasis with declining lung function.
- They have associated rheumatoid arthritis, immune deficiency, inflammatory bowel disease or primary ciliary dyskinesia.
- They have allergic bronchopulmonary aspergillosis.
- Patients with advanced disease and those considering lung transplantation.

Lower-risk patients can be routinely reviewed in primary care. The exact frequency of review should be determined in the initial treatment plan, in consultation with the respiratory specialist and also by changing clinical conditions, but should be at a minimum frequency of once a year.

Factors to be routinely monitored by the primary care team (see Fig. 2) include:

- Assessment of symptoms (cough, sputum production and breathlessness) and their impact on daily activities.
- Chronic cough that can have a significant effect on daily activities, including sexual relations and urinary incontinence in women.^16^
- Exacerbation frequency.
- Body mass index (BMI).
- Pulse oximetry.
- Spirometry.
- The sputum should be sent for microbiological analysis when the patient is clinically stable, and should be sent at the start of an exacerbation before antibiotic treatment is started.^1^
- Compliance with sputum clearance exercises and shared treatment plan.

Deterioration in lung function, exacerbation frequency or clinical state should prompt specialist referral (see above). If the patient’s BMI is <20 kg/m^2^, then specialist nutritional advice should be sought. In addition, the patient should be referred to a respiratory physiotherapist to reinforce the sputum clearance exercises if necessary. Similarly, referral for pulmonary rehabilitation should be considered, if the patient has functionally disabling breathlessness (MRC score ≥ 2).

Common co-morbidities, such as mild-to-moderate asthma or COPD, can be treated in the primary care setting, according to national or international guidelines for these conditions.^17–20^ It is recommended that chronic rhinosinusitis be treated with nasal saline douching and intranasal steroids. There is a strong association between bronchiectasis and gastro-oesophageal disease (GORD) and patients with GORD have a more severe disease.^21^ The guideline recommends that symptoms of GORD are specifically sought out and treated, according to the existing NICE guidance, but do not provide evidence that treatment of GORD will improve bronchiectasis outcomes.

## Management

**Fig. 3:**
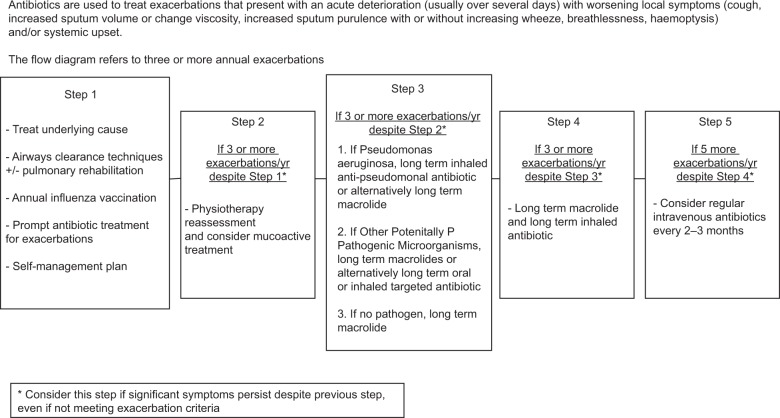
Stepwise management of patients with bronchiectasis. Reproduced from ref. ^1^, with permission from BMJ Publishing Group Ltd.

The overall aims of management are to prevent further damage (treat the underlying cause if possible), maximise function and quality of life and prevent and treat exacerbations. Figure 3 shows a stepwise approach to management.^1^

## Physiotherapy

All patients with bronchiectasis should be assessed by a respiratory physiotherapist. Chest physiotherapy can mobilise secretions and aid effective expectoration,^22–24^ providing improvement in cough scores. Table 3 shows the key elements of physiotherapy intervention.

**Table 3:** Key elements of physiotherapy in bronchiectasis

• Patients are taught to carry out airway clearance techniques, such as active cycle of breathing technique (ACBT)^15^ (or using an oscillating positive expiratory pressure device, such as the “Flutter” and “Acapella”.^16^ This is tailored to the individual patient (usually once to twice daily) and may be increased during an exacerbation.
• Airway clearance may be optimised using postural drainage (use of gravity-assisted positioning to drain areas of the lung) and prior inhalation of isotonic or hypertonic saline.
• Consider a 6-month trial of mucolytic agents (such as oral carbocisteine) if there are continued difficulties with sputum expectoration.
• Pulmonary rehabilitation should be offered where a patient is functionally limited by breathlessness (MRC score ≥ 2).^16^

## Influenza and pneumococcal immunisation

It is important to offer annual influenza immunisation to all patients with bronchiectasis.^25^ In addition, the guidelines recommend that polysaccharide pneumococcal vaccination should be offered to all patients with bronchiectasis (if not previously given), although this recommendation comes from evidence that 23 valent pneumococcal vaccinations can reduce the risk of all-cause pneumonia.^26^

## Inhaled corticosteroids and bronchodilators

Treatment with inhaled corticosteroids (ICS) in bronchiectasis is not recommended, unless the drug is needed for comorbid diseases, such as asthma or COPD, according to treatment guidelines.^17–20^ A Cochrane review showed that treatment with ICS demonstrated a minimal reduction in sputum volume, but no effect on exacerbations.^27^ The studies included in the article used a relatively high dose of ICS (≥ 800 mcg of beclometasone equivalent) and there was a high incidence of local and systemic side effects. It is recommended to offer a trial of long-acting inhaled bronchodilators in patients with significant breathlessness or where there is coexistent asthma and/or COPD.^1^

No evidence was found for the use of short-acting beta-2 agonists and limited evidence for long-acting beta-2 agonists (LABA) and long-acting anti-muscarinic agents (LAMA).^28^ Use of LABA and LAMA is recommended in patients with significant breathlessness and coexistent obstructive airway disease of whatever aetiology.

## Long-term antibiotic therapy

Long-term—3 months or more—antibiotic therapy should be considered for patients with three or more exacerbations per year, which should be initiated and monitored by specialist teams in secondary care. Treatment with oral azithromycin, 250 mg, three times weekly for a year can reduce the incidence of exacerbations in patients with bronchiectasis,^29^ and this is recommended as a pragmatic starting dose in the guidelines.^1^ In patients with concurrent *P. aeruginosa* infection, the recommended first-line therapy is inhaled colistin. Sputum samples from a secondary care population show that *H. influenzae* and *P. aeruginosa* are the most common bacterial isolates with other pathogens, such as *M. catarrhalis, S. pneumoniae* and non-tuberculous mycobacteria also being found.^30^ The presence of *P. aeruginosa* not only guides the choice of antibiotic therapy, but also signifies a worse prognosis with a threefold risk in mortality, higher risk of hospital admission and poorer quality of life.^31^

## Management of the acute exacerbation

Where an acute exacerbation incident is suspected, the first step should be to assess the vital signs (pulse oximetry, pulse rate, respiratory rate, temperature and blood pressure). In the primary care setting, the patient should be treated for acute co-morbidities, such as asthma and COPD, according to disease-specific guidelines.^17–20,32^ The patient should be considered for admission to a secondary care setting, where there are features of sepsis or respiratory distress, where there are significant co-morbidities and/or psychosocial factors or there is a need for intravenous antibiotics, where a community service to provide these is not available.

## Acute antibiotic therapy

Where possible, the sputum should be obtained for culture and sensitivity testing prior to commencing antibiotics. However, empirical antibiotics can be started whilst awaiting the sputum results. Table 4 shows common organisms associated with acute exacerbations of bronchiectasis and their treatment in primary care. In general, antibiotic courses for 14 days are standard and should always be used in patients infected with *P. aeruginosa*. Shorter courses may suffice in patients with mild bronchiectasis (usually 7–10 days). It is recommended to consider providing a home sputum pot and standby antibiotics for future exacerbations.

**Table 4:** Common organisms associated with acute exacerbations of bronchiectasis and suggested oral antibiotics^1^

Organism	Recommended first-line treatment	Length of treatment	Recommended second-line treatment	Length of treatment
*Streptococcus pneumoniae*	Amoxicillin 500 mg tds	14 days	Doxycycline 100 mg BD	14 days
*H. influenzae*-beta lactamase negative	Amoxicillin 500 mg tds or amoxicillin 1G tds or amoxicillin 3G BD	14 days	Doxycycline 100 mg BD or ciprofloxacin 500 or 750 mg BD or ceftriaxone 2G OD (IV)	14 days
*H. influenzae*-beta lactamase pos	Amoxicillin with clavulanic acid 625 one tablet tds	14 days	Doxycycline 100 mg BD or ciprofloxacin 500 or 750 mg BD or ceftriaxone 2G OD (IV)	14 days
*Moraxella catarrhalis*	Amoxicillin with clavulanic acid 625 one tablet tds	14 days	Clarithromycin 500 mg BD or doxycycline 100 mg BD or ciprofloxacin 500 or 750 mg BD	14 days
*Staphylococcus aureus* (methicillin sensitive)	Flucloxacillin 500 mg qds	14 days	Clarithromycin 500 mg BD or doxycycline 100 mg BD or amoxicillin with clavulanic acid 625 one tablet tds	14 days
*Pseudomonas aeruginosa*	Oral ciprofloxacin 500 mg BD (750 mg BD in more severe infection)	14 days	Discuss with a respiratory specialist/microbiologist	

BNF 72 (March 2017) *OD* once daily; *BD* twice daily; *TDS* three times a day; *IV* intravenous

## Prognosis

Patients with mild bronchiectasis should have a normal life expectancy, but in patients with more-advanced bronchiectasis, there is an increased risk of recurrent exacerbations, vascular disease and mortality.^30,31,33^

## Conclusions

A diagnosis of bronchiectasis should be suspected when a patient presents with a persistent or recurrent productive cough. Definitive diagnosis is made by thin-section CT scan of the chest. Assessment of bronchiectasis should be carried out in secondary/tertiary care, where a shared management plan should be formulated with the patient and the community/primary care team.

Many patients can be routinely managed in primary care, where the cornerstone of management is physiotherapy to improve sputum clearance and maximise exercise capacity and prompt treatment of exacerbations with antibiotics.

The British Thoracic Society Bronchiectasis Guidelines provide advice for diagnosis and management of primary care in the United Kingdom. Although some of the advice, such as the type of antibiotics to be used, is specific to the United Kingdom, many of the recommendations are in line with those of the European Respiratory Society Guidelines 2017,^34^ and provide a sound basis for primary care management of this increasingly prevalent disease throughout the world.

## Data Availability

This article draws on the BTS Guideline for Bronchiectasis in Adults—evidence tables supporting the guideline are available on the BTS website: https://www.brit-thoracic.org.uk/quality-improvement/guidelines/bronchiectasis-in-adults/.
